# Abbreviated MRI Protocol for the Assessment of Ablated Area in HCC Patients

**DOI:** 10.3390/ijerph18073598

**Published:** 2021-03-30

**Authors:** Vincenza Granata, Roberta Grassi, Roberta Fusco, Sergio Venanzio Setola, Andrea Belli, Mauro Piccirillo, Silvia Pradella, Marzia Giordano, Salvatore Cappabianca, Luca Brunese, Roberto Grassi, Antonella Petrillo, Francesco Izzo

**Affiliations:** 1 Radiology Division, “Istituto Nazionale Tumori IRCCS Fondazione Pascale—IRCCS di Napoli”, 80131 Naples, Italy; v.granata@istitutotumori.na.it (V.G.); s.setola@istitutotumori.na.it (S.V.S.); a.petrillo@istitutotumori.na.it (A.P.); 2 Division of Radiology, University of Campania Luigi Vanvitelli, 80127 Naples, Italy; robertagrassi89@gmail.com (R.G.); marziagior@gmail.com (M.G.); salvatore.cappabianca@unicampania.it (S.C.); roberto.grassi@unicampania.it (R.G.); 3 Hepatobiliary Surgical Oncology Division, “Istituto Nazionale Tumori IRCCS Fondazione Pascale—IRCCS di Napoli”, 80131 Naples, Italy; a.belli@istitutotumori.na.it (A.B.); mauro.piccirillo@istitutotumori.na.it (M.P.); f.izzo@istitutotumori.na.it (F.I.); 4 Division of Radiology, “Azienda Ospedaliera Universitaria Careggi”, 50139 Florence, Italy; pradellas@aou-careggi.toscana.it; 5 Radiology Division, Università degli Studi del Molise, Via Francesco De Sanctis, 86100 Campobasso, Italy; luca.brunese@unimol.it; 6 Fondazione SIRM, 20122 Milan, Italy

**Keywords:** HCC, ablation treatment, magnetic resonance imaging, DWI, contrast enhancement MRI

## Abstract

Background: Liver Imaging Reporting and Data Systems (LI-RADS) Treatment Response Algorithm (TRA) was created to provide a standardized assessment of hepatocellular carcinoma (HCC) following loco regional therapy. The aim of this study was to compare sensitivity of standard MRI protocol versus abbreviated protocol (only T1-Weigthed fat suppressed (FS) sequences pre- and post-contrast phase) in the detection of ablated area according to LI-RADS Treatment Response (LR-TR) categories. Methods: From January 2015 to June 2020, we selected 64 patients with HCC, who underwent Radiofrequency ablation (RFA) or Microwave ablation (MWA) treatment. According to inclusion criteria, 136 pathologically proven treated HCC (median 2, range 1–3 per patient; mean size 20.0 mm; range 15–30 mm) in 58 patients (26 women, 32 men; median age, 74 years; range, 62–83 years) comprised our study population. For each ablated area, abbreviated protocol, and standard Magnetic Resonance Imaging (MRI) studies were independently and blindly assessed in random order within and between three expert radiologists. Each radiologist assessed the ablated area by using the following categories: “LR-TR Non-viable” = 1; “LR-TR Equivocal” = 2 and “LR-TR Viable” = 0. Results: According to the concordance between MRI and Contrast enhancement ultrasound (CEUS) among 136 treated HCCs, 115 lesions were assessed as non-viable or totally ablate and 21 as viable or partially ablate. The accuracy for standard MRI protocol and abbreviated MRI protocol for predicting pathologic tumor viability of a consensus reading was 98.6% (sensitivity = 100%; specificity = 98.3%; positive predictive value = 91.3% and negative predictive value = 100%). No differences were found in sensitivity or specificity between standard MRI LR-TR viable and abbreviated MRI LR-TR viable categories (*p* value > 0.05 at McNemar test). Conclusion: The abbreviated dynamic protocol showed similar diagnostic accuracy to conventional MRI study in the assessment of treated HCCs, with a reduction of the acquisition study time of 30% respect to conventional MRI.

## 1. Introduction

Ablation treatment is a minimally invasive tool that is commonly employed in the hepatic primary or secondary liver tumors [1,2,3,4,5]. This treatment is considered a possible first-line tool in small hepatocarcinoma (HCC) or the best therapeutic choice for nonsurgical patients with early stage HCC [1]. Patients are required to have either a single tumor smaller than 5 cm or as many as three nodules smaller than 3 cm each and no evidence of vascular invasion or extrahepatic spread [1,3]. Tumor size is a prognostic aspect to expect the outcome of therapy. The target tumor should not exceed 3–4 cm in longest axis to ensure complete ablation with most of the currently available devices [2,3,4,5,6,7].

Among all the ablation therapy, radiofrequency ablation (RFA) is a frontline technique for HCCs smaller than 20 mm [1]. Several studies have assessed the efficacy of RFA compared to surgical procedure and have established that RFA is a safety and effective [5,8]. With the microwave (MW) technology progress and a continuously cooled electrode development, Microwave ablation (MWA) has recently been used more frequently in treatment of HCC [1]. In patients with HCC treated with MWA (compared with RFA), overall survival varies between 22 months for focal lesion >3 cm (vs. 21 months) and 50 months for focal lesion ≤ 3 cm (vs. 27 months), local recurrence between 5% (vs. 46.6%) and 17.8% (vs. 18.2%), complication rate between 2.2 % (vs. 0%) and 61.5% (vs. 45.4%), disease-free survival, between 14 months (vs. 10.5 months) and 22 months (vs. no data reported), and mortality between 0% (vs. 0%) and 15% (vs. 36%) [1].

Response evaluation of ablation therapy is problematic and is correlated to the type of them used. RFA and MWA are hyperthermic treatment that use energy to heat the target area to at least 60 °C [9]. In oncology, tumor response was initially measured according to the bi-dimensional World Health Organization (WHO) criteria and afterward according to the mono-dimensional Response Evaluation Criteria In Solid Tumors (RECIST) guidelines [10]. RECIST are unfitting to evaluate locoregional treatment, since the current morphologic response criteria do not offer the sufficient data to define the efficacy of therapy. Hence, response criteria dedicated to ablation therapies are needed in clinical practice, other than in clinical trials [10,11,12,13,14,15,16,17,18,19]. According to Lencioni et al., it is mandatory to obtain a dual-phase imaging of the liver, arterial and portal phase, while the equilibrium phase is useful but not necessary [11]. Additionally, Liver Imaging Reporting and Data Systems (LI-RADS) Treatment Response Algorithm (TRA) has been conceived to offer a standardized evaluation of HCC following loco regional therapy (LRT). LI-RADS TRA offers a step-by-step method to assess each nodule individually for precise treatment evaluation [20]. Although the adoption of imaging tool (computed tomography (CT) or magnetic resonance imaging (MRI)) can be subject to patient characteristics and institutional inclination, it is critical to maintain consistency in the imaging performed before and after treatment. Precise assessment of post-treatment imaging is critical to guide further management decisions and requires comparison of post- treatment with pre-treatment imaging to recognize the original lesion diameter and enhancement features [20]. Follow-up imaging of patients with treated HCC is performed to evaluate for new lesions, monitor for evidence of early recurrence and observe for neovascularity that may allow for detection of pathological angiogenesis within the ablation zone. The presence of an enhanced area and washout in a treated lesion raises the suspicion for local recurrence. However, the absence of wash-out does not exclude the suspicion of recurrence [10]. Earlier detection of these suspicious lesions would lend itself well to early retreatment. CT or MRI are considered the standard imaging modalities for evaluating therapeutic efficacy in the follow-up of the patient treated with ablative therapy, because of high diagnostic accuracy and large field of view which permits complete evaluation of the tumor and the whole liver parenchyma [10]. CEUS is recommended in the post-treatment follow-up when CT and MRI are inconclusive or contraindicated. However, CEUS can be utilized as a method for secondary surveillance, normally performed every 3–4 months, allowing for an early detection of the recurrence, while still screening via intermittent CE- CT or CE-MRI along with clinical status, biochemical liver function tests, and AFP serum level [10].

LI-RADS TRA is formed after mRECIST, and it primarily depend on post treatment arterial phase hyper enhancement (APHE) to identify viable tumor. Additionally, LI-RADS TRA is unique. In fact, in addition to APHE, the classification of viable lesion includes washout appearance or enhancement similar to that seen before therapy. During image analysis of treated HCC, each liver observation should be reported separately according to the LI-RADS Treatment Response (LR-TR) categories [21]. Treatment response categories include: “LR-TR Non-viable”, “LR-TR Equivocal”, and “LR-TR Viable”. In instances where the technical limitation precludes characterization of the tumor, an “LR- TR Nonvaluable” category can be assigned [21].

The aim of this study was to compare sensitivity of standard MRI protocol versus abbreviated protocol (only T1-W fat suppressed (FS) sequences pre- and post-contrast phase) in the detection of ablated area according to LR-TR categories. The gold standard was concordance between MR LR-TR categories with contrast enhancing ultrasound (CEUS) data.

## 2. Methods

### 2.1. Study Population

Institutional review board of National Cancer Institute of Naples approved this retrospective study, and the patient’s informed consent requirement has been waived. From January 2015 to June 2020, we selected 64 patients with HCC, who underwent RF or MWA treatment. The inclusion criteria for the study population were as follows: (a) patients with radiological diagnosis of HCC; (b) patients who had subjects to MRI study within one month before treatment and who underwent MRI post 1 month after treatment; (c) patients who had less than a one-month between radiological and pathological diagnosis and (d) patients with pathologically proven HCC. The exclusion criteria were as follows: (a) divergence between radiological and pathological diagnosis and (b) no accessible MR study pre- or post-treatment.

In total, 58 patients with treated HCC confirmed at pathological analysis satisfied the inclusion criteria. Three patients were excluded because MRI studies post treatment were no accessible and 3 because there was divergence between radiological and pathological diagnosis. Finally, 136 pathologically proven (51 well, 48 moderately, and 37 poorly differentiated) treated HCC (median 2, range 1–3 per patient; mean size 20.0 mm; range 15–30 mm) in 58 patients (26 women-32 men; median age, 74 years; range, 62–83 years) comprised our study population. Characteristics of the 58 patients are summarized in Table 1.

### 2.2. MRI Protocol

MR studies was performed with a 1.5 T scanner (Magnetom Symphony, with Total Imaging Matrix Package, Siemens, Erlangen, Germany) with an eight-element body coil and a phased array coil. Comprehensive data on MR parameters is reported in Table 2. A non-specific agent the Gd-BT-DO3A (Gadovist, Bayer Schering Pharma, Berlin, Germany) was employed, according our department guidelines. All patients received 0.1 mL/kg of Gd-BT-DO3A by means of a power injector (Spectris Solaris^®^ EP MR, MEDRAD Inc., Indianola, IA, USA), at an infusion rate of 2 mL/s. Arterial phase was acquired 7 s after contrast agent arrival at the thoracic aorta by using a fluoroscopic monitoring system. After contrast medium injection portal and equilibrium phase were obtained 60 s and 3 min after, respectively.

### 2.3. Contrast Enhancement Ultrasound Protocol

For each ablated area, abbreviated protocol and standard MR studies were independently and blindly assessed in random order within and between three expert radiologists. To reduce recall bias, all three readers maintained an interval of more than two weeks between interpretation sessions of abbreviated protocol and standard MR study. Each radiologist assessed the ablated area by using the following categories: “LR-TR Non-viable” = 1; “LR-TR Equivocal” = 2 and “LR-TR Viable” = 0. In instances where technical limitation precludes characterization of the tumor, an “LR- TR Nonvaluable” = 0 category can be assigned [21]. The radiologists evaluated the following data for MR conventional studies: signal intensity (SI) on T1- and T2-weighted (W) images, SI on DWI sequences and the ADC map, vascular enhancement pattern during arterial, portal, and equilibrium phase.

The radiologists evaluated the following data for MR abbreviated protocol: signal intensity (SI) on T1-W pre contrast study and vascular enhancement pattern during arterial, portal and equilibrium phase.

The SI of the treated area was described as isointense, hypointense, and hyperintense related to nearby hepatic parenchyma. The diffusion-weighted signal decay was assessed by the mono-exponential model, according to the equation ADC = (ln (S0/Sb))/b, where Sb is the SI with diffusion weighting b and S0 is the non-diffusion-weighted signal intensity. This analysis was region of interest (ROI) based using median value of single voxel signals for each b value. ROIs for the tumor were manually drawn to include such hyperintense voxels on image at b value 800 s/mm^2^.

The enhancement pattern during arterial-, portal-, and equilibrium phase was described according to LI-RADS TR features [21].

The gold standard was concordance between MR LR-TR categories with contrast enhancing ultrasound (CEUS) data. CEUS was performed using sulfur hexaflouride microbubbles (SonoVue^®^, Bracco, Italy) before (median: seven days; range: 1–15 days) and post ablation procedures (median: seven days; range: 1–15 days) to assess the therapeutic result. The most important imaging finding that suggests complete treatment of a focal liver tumor is the disappearance of any previously visualized vascular enhancement on contrast- enhanced images. Pre-treatment images and/or movie clips were digitally stored to be compared with post-ablation study. The assessment of viable or non-viable treated area is based on the visual assessment of tumor viability defined as nodular, mass-like, or thick, irregular tissue in or along the treated lesion showing APHE or washout appearance, or an enhancement similar to that observed before treatment.

### 2.4. Statistical Analysis

The diagnostic performances of viable categories in MRI-standard LR-TR and MRI-abbreviated TR for the total study sample were compared for each reviewer and in a consensus reading with the McNemar test to investigate the differences based on diagnostic protocol, with the CEUS results for tumor viability used as the reference standard. Sensitivity, specificity, positive and negative predictive value (PPV and NPV), and accuracy (ACC) were calculated.

Chi square test with Yates’s correction was applied to identify the MRI imaging feature significant to predict tumor viability and nonviability considering the prevalence rate in viable and in non-viable lesions.

The assessment of observer variability for the three readers to assign the category was performed by calculating the intraclass correlation coefficient.

All analyses were performed using Statistics Toolbox of Matlab R2007a (The Math-Works Inc., Natick, MA, USA).

## 3. Results

According to the reference standard among 136 HCCs treated with RF or MWA, 115 lesions were assessed as non-viable or totally ablate and 21 as viable or partially ablate.

According to both MR protocols, 113 lesions were assessed as non-viable and 23 as viable.

**Figure 1:**
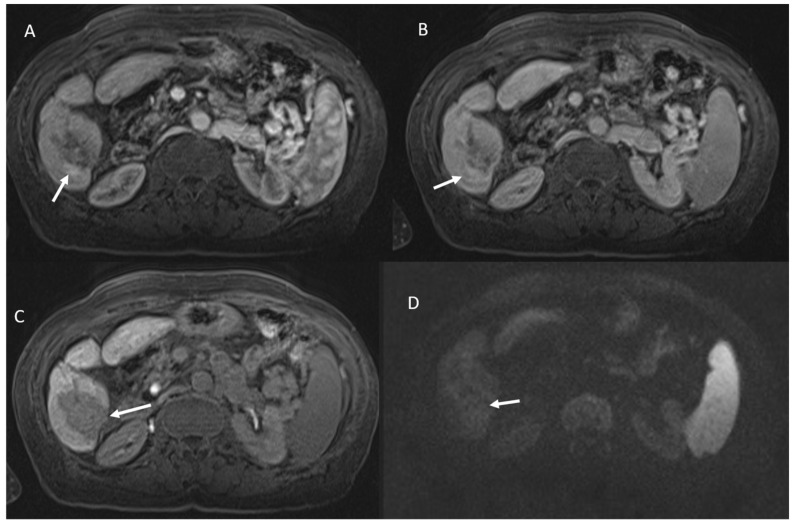
Vascular shunt in HCC treated patient. During contrast the arrow shows APHE in arterial phase (**A**), without wash-out appearance in portal phase (**B**). Ablated area (arrow) in hepatobiliary phase (**C**) without restricted diffusion (**D**).

The two lesions that were erroneously defined as viable presented peripherally an area of vascular shunt (Figure 1).

**Figure 2:**
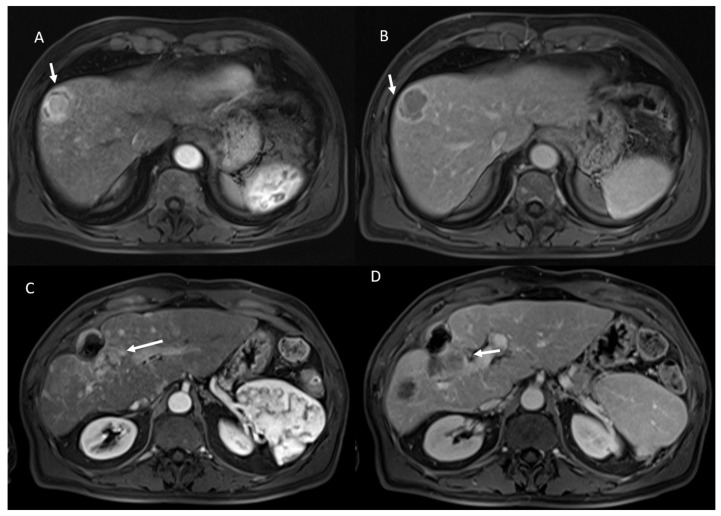
HCC on VIII hepatic segment: arrow shows APHE in arterial phase (**A**), with wash out and capsule appearance in portal phase (**B**). Post-treatment MRI: arrow shows APHE (**C**) and wash-out appearance in portal phase (**D**) in viable lesions.

**Figure 3:**
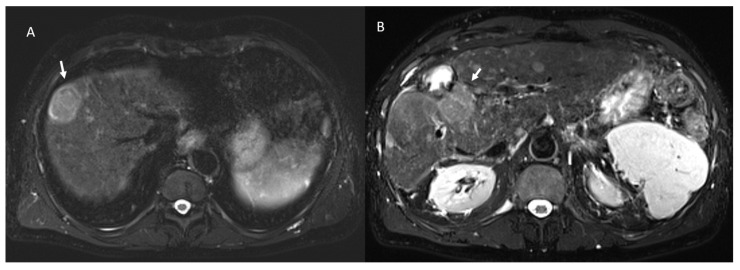
The same patient of Figure 2: in pre-treatment SPACE FS T2-W sequence (**A**), the lesion shows hyperintese SI; in post-treatment SPACE FS T2-W sequence (**B**), viable lesion shows hypeintense SI.

**Figure 4:**
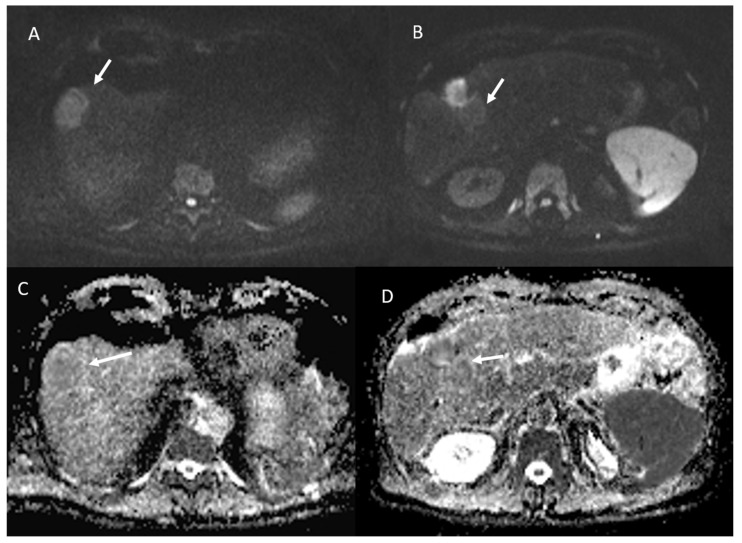
The same patient of Figure 2 and Figure 3: in pre-treatment b800 s/mm^2^ (**A**), the lesion shows restricted diffusion with isointense SI in ADC map (**C**); in post-treatment b800 s/mm^2^ (**B**), viable lesion shows restricted diffusion with iso-hypointense S. in ADC map (**D**).

Among the 23 viable lesions:

- 21 showed APHE and 2 rim APHE during arterial Phase (Figure 2).
- 23 showed wash-out appearance during portal phase and hypointense SI in equilibrium phase (Figure 2).
- 23 lesions were hyperintense in T2-W and hypointense in T1-W sequences (Figure 3).
- 23 lesions showed restricted diffusion with hypointense SI in ADC map (Figure 4).

**Figure 5:**
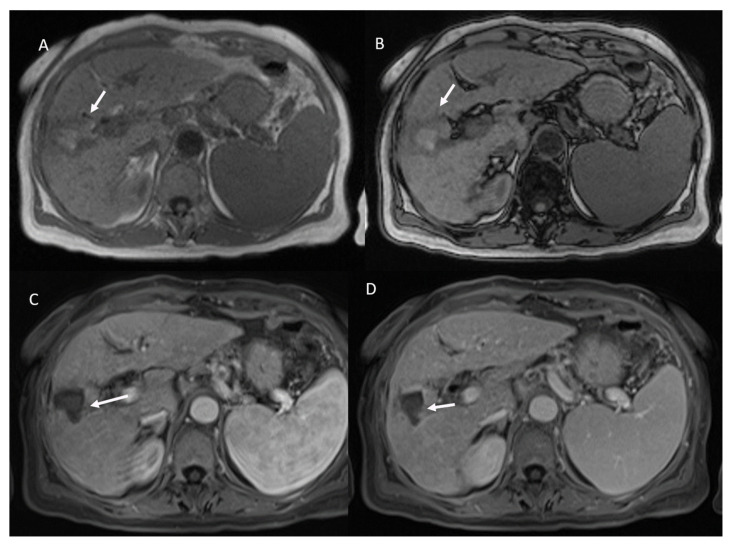
Non-viable treated HCC. In T1-W sequences (**A**, in phase and **B**, out of phase) the arrow shows hyperintense SI of ablated area. During contrast study the non-viable lesion shows hypointense SI in arterial (**C**) and portal (**D**) phase.

**Figure 6:**
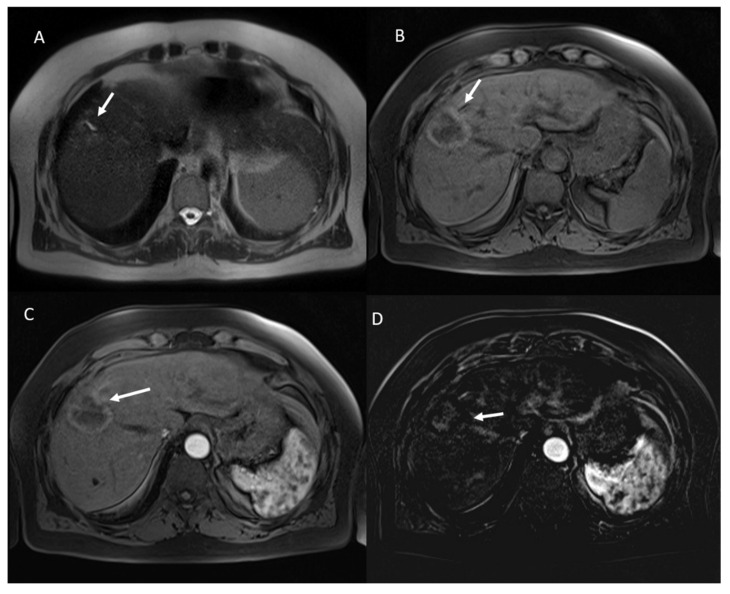
Non-viable treated HCC. In T2-W sequences (**A**) the arrow shows isointense SI of ablated area, with targetoid appearance in T1-W sequence, due to peripheral hypintense rim (**B**). Post-contrast arterial phase (**C**) analysis in subtraction (**D**) shows no APHE.

**Figure 7:**
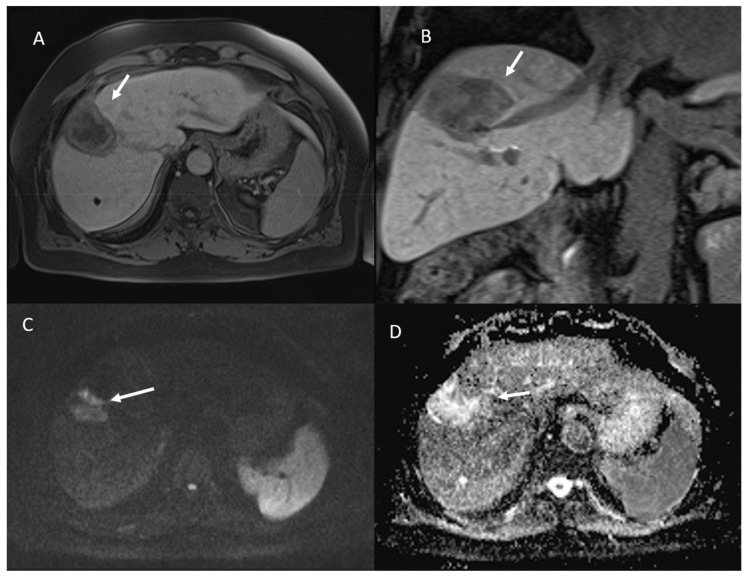
The same patient of Figure 6. Non-viable lesion shows targetoid appearance in the portal (**A**) phase of contrast study and in the hepatospecific phase (**B**), with restricted diffusion in b800 s/mm^2^ (**C**) and hyperintense SI in ADC map (**D**).

Among 113 non-viable lesions:

- 53 showed non rim APHE and 60 hypointense SI during arterial phase (Figure 5).
- 3 showed peripheral washout appearance and 110 hypointense SI in portal phase (Figure 5).
- 113 lesions were hypointense in equilibrium phase.
- 98 showed iso-hypointense SI in T2-W and 15 iso-hyperintense SI in T2-W (Figure 6).
- 100 showed hyperintense SI in T1-W and 113 targetoid appearance.
- 84 lesions showed restricted diffusion (Figure 7) with iso-hypointense in ADC map and 29 showed no restricted diffusion.

### 3.1. Category Assignment According to MR

For reviewer 1, 113 lesions were assigned to the non-viable category and 23 to the viable category based on the standard MRI protocol and abbreviated MRI protocol.

For reviewer 2, 109 lesions were assigned to the non-viable category, 3 to the equivocal category and 24 to the viable category based on the abbreviated MRI protocol; otherwise, based on standard protocol, 113 lesions were assigned to the non-viable category, two to the equivocal category, and 21 to the viable category.

For reviewer 3, 111 lesions were assigned to the non-viable category, two to the equivocal category, and 23 to the viable category based on the abbreviated MRI protocol; otherwise, based on standard protocol, 113 lesions were assigned to the non-viable category and 23 to the viable category.

For reviewers 2 and 3, the equivocal category was assigned the least based on DWI assessment.

For category assignment, the ICC was 0.9 among classification provided by three radiologists for standard protocol and was 0.95 among classification provided by three radiologists for abbreviated protocol.

### 3.2. Diagnostic Performance of Viable Category

Diagnostic performance for each reader and for consensus reading was reported in Table 3.

The accuracy for both standard MR protocol and abbreviated MR protocol for predicting pathologic tumor viability of a consensus reading was 98.6% (sensitivity = 100%; specificity = 98.3%; positive predictive value = 91.3% and negative predictive value = 100%). No differences were found in sensitivity or specificity between standard MR LR-TR viable and abbreviated MRI LR-TR viable categories (*p* value > 0.05 with a McNemar test).

### 3.3. Imaging Features for the Prediction of Tumor Viability

Table 4 reports the prevalence of Imaging Features at MRI in viable and non-viable lesions. Among MRI features of LR-TR imaging criteria in the consensus reading, APHE (prevalence rate = 91.3%), wash-out (prevalence rate = 100.0%) appearance, hyperintense in T2-W (prevalence rate = 100.0%), hypointense in T1-W (prevalence rate = 100.0%) showed the highest prevalence rate in viable lesions compared to non-viable lesions with difference statistically significant (*p* value < 0.05 with a chi-square test).

Therefore, for abbreviated protocol, among MRI features of LR-TR imaging criteria in the consensus reading, APHE, wash-out appearance and hypointense in T1-W were the most prevalence features in viable lesions (*p* value < 0.05 with a chi-square test).

### 3.4. Imaging Features for Prediction of Tumor Non-Viability

Table 4 reports the prevalence of imaging features with MRI in viable and non-viable lesions. Among MRI features of LR-TR imaging criteria in the consensus reading, hypointense SI in portal phase (prevalence rate = 97.3%), iso-hypointense SI in T2-W (prevalence rate = 86.7%), hyperintense SI in T1-W (prevalence rate = 88.5%), targetoid appearance in T1-W (prevalence rate = 100.0%) showed the highest prevalence rate in non-viable lesions compared to viable lesions with difference statistically significant (*p* value < 0.05 with a chi-square test).

Therefore, for abbreviated protocol, among MRI features of LR-TR imaging criteria in the consensus reading, hypointense SI in portal phase, hyperintense SI in T1-W and targetoid appearance in T1-W (prevalence rate = 100.0%) were the most prevalence features in non-viable lesions (*p* value < 0.05 with a chi-square test).

## 4. Discussion

Although the efficacy of MRI in HCC patients, both for detection and staging and for evaluation after treatment is well established [22,23,24,25], its high cost and longer study time compared with CT might limit its common application. Consequently, several studies assessed abbreviated MRI protocols for HCC patient screening [26,27,28,29,30,31,32,33,34]. Three methods have been improved: non-contrast abbreviated MRI (NC-AMRI), dynamic contrast-enhanced abbreviated MRI, and hepatobiliary phase contrast-enhanced (HBP) abbreviated MRI. These approaches can be concluded in nearly ten minutes or less, significantly less if compared to conventional MRI protocol.

NC-AMRI presents several benefits. By avoiding gadolinium-based contrast agent (GBCA) administration, it limits costs, is safe, avoiding IV placement, reduces acquisition time simplifying workflow. The main limit of NC-AMRI is that it is based completely on unenhanced study, diminishing the detection of HCC as compared to post contrast sequences used in the other abbreviated approaches. The addition of DWI could facilitate the assessment of liver nodules. Nevertheless, DWI is challenging and often suffers from a variety of artefacts, that can cause blind spots, most often near the liver dome or in the left lobe. Many early stage HCCs may not exhibit restricted diffusion relative to liver [24].

Dynamic contrast-enhanced AMRI (Dynamic-AMRI) acquires dynamic contrast enhanced images using T1-W sequences with fat suppression following administration of an extracellular contrast medium. The dynamic component describes the images acquisition at pre-determined and successive phases to identify and characterize HCCs based on the vascular pattern [35]. Dynamic-AMRI offers the advantages of to define major features of HCC according to LI-RADS so that dynamic AMRI alone is sufficient to definitive diagnosis of HCC. Additionally, it offers cost benefits, since the contrast agents used in dynamic protocol are less expensive than the contrast agent (gadoxetate disodium) required for HEPATOBILARY-PHASE (HBP)-AMRI. The disadvantages of this approach is related to the lack of additional non-contrast sequences, which may give ancillary features [35].

HBP-AMRI is based on the acquisition of T1-W FS sequences after the administration of the hepatobiliary agent, gadoxetate disodium. HBP-AMRI offers several advantages: high- contrast-to-noise, aiding in lesion detection. The 20-min delay also allows hand injection of contrast while the patient is in the waiting room, which simplifies workflow, reduces the time the patient is on the MRI scanner, thus reducing the examination cost. Finally, HBP-AMRI are interpreted using a simple score system obtained by LI-RADS US surveillance [36]. The limits of HBP-AMRI are related to the contrast medium used. In fact, gadoxetate, is more expensive than the extracellular agents. Additionally, patients with advanced cirrhosis may have reduced hepatic function, which may limit contrast uptake, or may have areas of confluent fibrosis, which may reduce the accuracy for HCC detection by obscuring tumors (false negatives) or being mistaken for tumors (false positives) [26].

The concept of abbreviated MRI is not new. The greatest body of work and adoption of abbreviated MRI has been in the area of the screening, which shows benefits in diagnosis and resource use. These abbreviated MRI protocols are simplified shorter protocols comprising a small number of sequences that are tailored to evaluate a particular disease, and therefore, are less time intensive to perform and less laborious to interpret. In the context of HCC surveillance, so as during treatment assessment, multiphase abdominal MRI may take approximately 40 min to complete and US may take approximately 30 min; whereas, an abbreviated MRI protocol is typically performed in 15 min or less and includes only the sequences necessary for detection of HCC [26]. In addition, despite the diagnostic advantages of multi-phase contrast-enhanced CT and MRI, there are challenges and drawbacks that prevent their widespread use for surveillance. CT is associated with the risks of ionizing radiation exposure and adverse events related to iodinated contrast agents. Due to the repetitive nature of surveillance imaging, the use of CT results in an unacceptably high cumulative radiation risk, especially in patients with HBV infection and well-compensated cirrhosis, who have longer disease courses. Due to the large number of imaging sequences and the length and complexity of complete diagnostic MRI protocols, they are not cost- or time-effective for HCC surveillance. In recent years, with improvements in MRI technology and a focus on value in radiology, several investigators have suggested abbreviated MRI strategies in an effort to make MRI a more feasible option for clinical HCC surveillance [26].

To the best of our knowledge, it is the first study that assessed the role of abbreviated protocol, including a Dynamic- MRI, in treated HCC patients. We showed that the accuracy for both standard MR protocol and abbreviated MR protocol for predicting pathologic tumor viability of a consensus reading was 98.6% (sensitivity = 100%; specificity = 98.3%; positive predictive value = 91.3% and negative predictive value = 100%). No differences were found in sensitivity or specificity between standard MR LR-TR viable and abbreviated MRI LR-TR viable categories (*p* value > 0.05 with a McNemar test). Among MRI features of LR-TR imaging criteria in the consensus reading, APHE (prevalence rate = 91.3%), wash-out (prevalence rate = 100.0%) appearance, hyperintense in T2-W (prevalence rate = 100.0%), hypointense in T1-W (prevalence rate = 100.0%) showed the highest prevalence rate in viable lesions compared to non-viable lesions with difference statistically significant (*p* value < 0.05 with a chi-square test). Therefore, for abbreviated protocol, among MRI features of LR-TR imaging criteria in the consensus reading, APHE, wash-out appearance and hypointense in T1-W were the most prevalence features in viable lesions (*p* value < 0.05 with a chi-square test).

In our study, the choice of a dynamic protocol is linked to the criteria that define the effectiveness of a locoregional treatment [21], so we believe that it is necessary to use the contrast medium and, considering the limits of the EOB, an interstitial type [14]. To obtain an efficacy treatment is critical the creation of a rim of greater than 5–10 mm around the lesion; thus, an ablation area larger than the original lesion is a needed feature. Furthermore, the treated area should not exhibit residual enhancement. However, coagulation necrosis and cell death within the target can result in the development of a central zone of hyper-intense SI on the pre-contrast T1-W sequences. Consequently, subtraction images are essential to avoid interpreting these imaging characteristics as areas of APHE. Imaging findings suggestive of residual viable tumor are thick peripheral irregular nodular APHE with or without washout appearance, “washout” alone, enhancement characteristics similar to pre-treatment lesions, or discontinuity in the smooth thin peripheral rim of enhancement. Since, all these features can be assessed only with the use of contrast medium, an abbreviated protocol in the evaluation of the efficacy of an ablative treatment can only be a dynamic protocol. Our data showed no significant difference in diagnostic accuracy of standard and abbreviated MR studies. We found a high concordance of abbreviated and conventional study.

There are several considerations of abbreviated protocol that have to be done. First the economic affects in terms of reduction of acquisition study time and in terms of exam cost. The abbreviated MRI acquisition time is almost ten minutes, with a decrease of 30% respect to conventional MRI. Moreover, a substantial shortening of examination time will render MRI studies more acceptable for patients with claustrophobia. Additionally, it offers cost benefits, since the contrast medium used in dynamic protocol are typically less expensive than the hepatobiliary agent. Future evaluations are required to describe the cost-effectiveness of these protocols.

However, it should be said that a treated patient is a patient at risk for the development of new HCCs. Consequently, the dynamic sequences alone may not be concluded in the characterization of new nodules, as in the case of early HCC or dysplastic nodules. In fact, the limits of dynamic-AMRI are correlated to the absence of non-contrast sequences, which may provide ancillary features. The incapacity of dynamic abbreviated protocol to assess these features may cause mis-categorization of observations. In particular, dynamic-AMRI might over-categorize some vascular pseudo lesions (e.g., arterio-portal shunts) as indeterminate (LR-3), potentially leading to unnecessarily close follow up. In theory, dynamic-AMRI also might under categorize some early or small HCCs as LR-3, potentially delaying diagnosis [21].

Our study is not without limitations. First, the readers implicated in this study were expert radiologist, with an annual case load of almost 1000 liver MRI studies per year. Second, the quality of the images obtained using a state-of-the-art scanner was optimal. Therefore, our results are not directly applicable to other lower-volume non expert centers.

## 5. Conclusions

Abbreviated dynamic protocol showed similar diagnostic accuracy to the conventional protocol in the assessment of treated HCCs, with a reduction of the acquisition study time approximately of 30% respect to conventional MRI. However, our results are related to higher-volume expert liver centers.

## Figures and Tables

**Table 1:** Patients’ characteristics.

Description	Numbers (%)/Range
HCCs patients	58
Gender	Men 32 (55.2%)
	Women 26 (44.8%)
Age	74 years; range, 62–83 years
Number of hepatic nodules	136 HCCs (51 well, 48 moderately, and 37 poorly differentiated)
Single nodule	10 patients
Multiple nodules	48 (2 nodules in 18 patients and 3 in 30 patients)
Nodule size (mm)	mean size 20.0 mm; range 15–30 mm
Risk factor for liver cirrhosis	58 (100%)
Chronic hepatitis B	32 (55.2%)
Chronic hepatitis C	26 (44.8%)
Alcoholic liver cirrhosis	0%
Child–Pugh Classification	
A	58 (100%)
B	0%
Treatment	
RFA	36 patients (98 HCCs)
MWA	22 patients (38 HCCs)

Note. HCC = hepatocellular carcinoma; RFA: radiofrequency ablation; MWA: microwave ablation.

**Table 2:** MR acquisition protocol.

Sequence	Orientation	TR/TE/FA (ms/ms/deg.)	AT (min)	Acquisition Matrix	ST/Gap (mm)	FS
Trufisp T2-W	Coronal	4.30/2.15/80	0.46	512 × 512	4/0	without
HASTE T2-W	Axial	1500/90/170	0.36	320 × 320	5/0	Without and with (SPAIR)
HASTE T2-W	Coronal	1500/92/170	0.38	320 × 320	5/0	without
SPACE T2-W FS	Axial	4471/259/120	4.20	384 × 450	3/0	With (SPAIR)
In-Out phase T1-W	Axial	160/2.35/70	0.33	256 × 192	5/0	without
DWI	Axial	7500/91/90	7	192 × 192	3/0	without
VibeT1-W	Axial	4.80/1.76/12	0.18	320 × 260	3/0	with (SPAIR)

Note. Trufisp = True fast imaging with steady state precession; T2-W = T2-wegthed; T1-W = T1-weigthed; HASTE = HAlf fourier Single- shot Turbo spin-Echo; DWI = diffusion weigthed imaging; T1-W = T1-wegthed; FS = fat sat; VIBE = volumetric interpolated breath-hold; SPAIR = SPectral Attenuated Inversion Recovery.

**Table 3:** Diagnostic performance for each reader and for consensus reading.

Reader	MR Protocol	Sensitivity	Specificity	PPV	NPV	ACC
**Reviewer 1**	Standard MRI protocol	100.00	98.26	91.30	100.00	97.10
	Abbreviated MRI protocol	100.00	98.26	91.30	100.00	97.10
**Reviewer 2**	Standard MRI protocol	100.00	94.78	77.78	100.00	94.20
	Abbreviated MRI protocol	100.00	98.26	91.30	100.00	97.10
**Reviewer 3**	Standard MRI protocol	100.00	96.52	84.00	100.00	95.65
	Abbreviated MRI protocol	100.00	98.26	91.30	100.00	97.10
**Consensus**	Standard MRI protocol	100.00	98.26	91.30	100.00	97.10
	Abbreviated MRI protocol	100.00	98.26	91.30	100.00	97.10

Note. PPV = positive predictive value; NPV = negative predictive value; ACC = accuracy; MR = magnetic resonance.

**Table 4:** Prevalence of imaging features at MRI in viable and non-viable lesions according to conventional and abbreviated studies.

Imaging Features at MRI	Viable Lesions (n. 23)	Non-Viable Lesions (n. 113)	*p* Value with a Chi-Square Test
APHE	21/23 (91.3%)	0/113 (0.0%)	**0.03**
rim APHE	2/23 (8.7%)	53/113 (46.9%)	**0.001**
hypointense SI during arterial phase	0/23 (0.0%)	60/113 (53.1%)	**<<0.001**
wash-out	23/23 (100.0%)	3/113 (2.7%)	**<<0.001**
hypointense SI in equilibrium phase	23/23 (100.0%)	113/113 (100%)	0.9
hypointense SI in portal phase	0/23 (0.0%)	110/113 (97.3%)	**<<0.001**
hyperintese in T2-W	23/23 (100.0%)	0/113 (0.0%)	**<<0.001**
iso-hyperintense SI in T2-W	0/23 (0.0%)	15/113 (13.3%)	0.1
iso-hypointense SI in T2-W	0/23 (0.0%)	98/113 (86.7%)	**<<0.001**
hypointense in T1-W	23/23 (100.0%)	0/113 (0.0%)	**<<0.001**
hyperintense SI in T1-W	0/23 (0.0%)	100/113 (88.5%)	**<<0.001**
targetoid appearance in T1-W	0/23 (0.0%)	113/113 (100%)	**<<0.001**
restricted diffusion	23/23 (100.0%)	84/113 (74.3%)	0.1

Note. In bold were identified the imaging features at MRI with significant difference between the viable and non-viable group. APHE = arterial phase hyper enhancement; SI = signal intensity; T1-W = T1-wegthed; T2-W = T2-wegthed

## Data Availability

Not applicable.
